# Future detection and monitoring of diabetes may entail analysis of both β-cell function and volume: How markers of β-cell loss may assist

**DOI:** 10.1186/1479-5876-10-214

**Published:** 2012-10-30

**Authors:** Anita V Neutzsky-Wulff, Kim V Andreassen, Sara T Hjuler, Michael Feigh, Anne-Christine Bay-Jensen, Qinlong Zheng, Kim Henriksen, Morten A Karsdal

**Affiliations:** 1 Nordic Bioscience A/S, Herlev Hovedgade 207, DK-2730, Herlev, Denmark; 2 Nordic Bioscience A/S, Beijing, China

**Keywords:** Neo-epitope, Biomarkers, Type II diabetes mellitus, β-cell death, BIPED classification, Patient segregation, Personalized treatment

## Abstract

Disease heterogeneity is as major issue in Type II Diabetes Mellitus (T2DM), and this patient inter-variability might not be sufficiently reflected by measurements of glycated haemoglobin (HbA1c).

Β-cell dysfunction and β-cell death are initiating factors in development of T2DM. In fact, β-cells are known vanish prior to the development of T2DM, and autopsy of overt T2DM patients have shown a 60% reduction in β-cell mass.

As the decline in β-cell function and mass have been proven to be pathological traits in T2DM, methods for evaluating β-cell loss is becoming of more interest. However, evaluation of β-cell death or loss is currently invasive and unattainable for the vast majority of diabetes patients. Serological markers, reflecting β-cell loss would be advantageous to detect and monitor progression of T2DM. Biomarkers with such capacities could be neo-epitopes of proteins with high β-cell specificity containing post translational modifications. Such tools may segregate T2DM patients into more appropriate treatment groups, based on their β-cell status, which is currently not possible. Presently individuals presenting with adequately elevated levels of both insulin and glucose are classified as T2DM patients, while an important subdivision of those is pending, namely those patients with sufficient β-cell capacity and those without. This may warrant two very different treatment options and patient care paths.

Serological biomarkers reflecting β-cell health status may also assist development of new drugs for T2DM and aid physicians in better characterization of individual patients and tailor individual treatments and patient care protocols.

## Introduction

Type II Diabetes Mellitus (T2DM) is a heterogeneous type of disease [1], meaning that affected patients display a large variability with regards to disease characteristics. This patient heterogeneity is not sufficiently illustrated by the currently available and gold standard biomarkers for evaluation of T2DM: Fasting plasma glucose (FPG), oral glucose tolerance test (OGTT) and glycated haemoglobin A1c (HbA1c). These markers efficiently reflect blood glucose status, but they cannot characterize T2DM patients at a more detailed level, for example with regards to pancreatic function, peripheral effects of insulin and progression of secondary complications of the disease.

Hence novel biomarkers with the potential to segregate different T2DM patient subtypes will be imperative to deliver the best possible treatment to the individual patient in the future. A new panel of diabetes biomarkers is needed to identify and characterize these future patient subtypes and assign appropriate subtype treatments. Also drug development of novel T2DM treatments would benefit from biomarkers contributing with more elaborate pathological information.

Obvious candidates in a novel biomarker panel are those reflecting the health or death of β-cells [2]. β-cells are the focal point in both Type I Diabetes Mellitus (T1DM) and T2DM, because of their capacity to produce and secrete insulin. In both types of diabetes, β-cells are lost [3,4]. The vast majority of T1DM patients display a dramatic loss of β-cells as a consequence of a cell-mediated autoimmune attack on the β-cells, with consequent insulin deficiency and chronic hyperglycaemia [3]. At time of diagnosis, T1DM patients have had their β-cell mass reduced by 70-80% [5]. Patients suffering from T1DM are often lean and display a healthy phenotype when their insulin deficiency is properly treated. This is in contrast to T2DM patients, who often are overweight or obese and display a metabolic phenotype, which complicates the overall pathology. Loss of β-cells is an initiating event in development of T2DM and may occur gradually and long before the disease is diagnosed [4]. A 40% reduction in β-cell mass has been observed in obese individuals with impaired fasting glucose (IFG), and a 60% reduction has been observed in patients suffering from overt T2DM [4,6]. Furthermore, a strong correlation between decline in β-cell area and development of T2DM has been established in patients suffering from pancreatic disorders [7]. These findings clearly indicate that when the β-cell mass declines considerably, the body can no longer uphold normal glucose regulation, with development of T2DM as consequence.

It is often not well established how current and novel drugs for management of T2DM affect the β-cells at a more detailed level, despite the apparent need for improving this aspect of the disease. Biomarkers reflecting β-cell death and β-cell mass could aid in evaluation of positive and/or negative effects on β-cells inflicted by different types of drugs, and novel β-cell biomarkers could therefore be advantageous in future drug development programs.

The ability to detect β-cell loss could facilitate prognosis of disease development of T2DM at a much earlier point in time than any of the currently used diabetes biomarkers such as FPG, OGTT and HbA1c. In addition, different sub-groups of T2DM patients are highly likely to lose β-cells at different rates, and these differences may require different treatment regimens for individual patients [7]. Serological biomarkers reflecting β-cell death and β-cell volume would therefore be valuable tools for disease prognosis, early diagnosis and tailoring treatment to the individual.

Today, evaluations of β-cell mass are only rarely performed, as this currently requires obtaining biopsies, which is an invasive procedure to the patient. Post-mortem histological evaluations of β-cell mass can be performed, but these examinations will not elucidate decline in β-cell mass during disease. Furthermore, histological assessment of β-cell death is complicated by the fact that dead cells are rapidly removed from the islets by macrophages [4]. Serological biomarkers reflecting β-cell mass or β-cell death have the strong advantage of being non-invasive, and such markers could, like HbA1c, be used as continuous evaluation of disease development and disease management.

Potential serological biomarkers reflecting β-loss could be specific neo-epitopes of β-cell related proteins. When β-cells are being lost by apoptosis or necrosis [5], site-specific cleavage of β-cell specific proteins is expected to arise from cleavage by particular proteases, creating neo-epitopes for measurement in serum. By this mechanism a particular protein fingerprint is generated, and investigations of this specific protein fingerprint could provide information about pathological processes [8]. Measurements of neo-epitopes have in several diseases been shown to correlate with disease pathology [9-15], as generation of specific neo-epitopes arise from specific pathological processes [8,16,17]. An incomplete list of β-cell proteins that might be of interest as novel biomarkers could include insulin, amylin, incretin receptors, special neuronal proteins and proteins which act as autoantigens in T1DM.

In the current review we describe how new biomarkers reflecting β-cell loss are desired to better identify people at risk of developing T2DM, to diagnose the disease earlier than is possible with current biomarkers, to better characterize affected patients, to optimize individual treatment, and to evaluate the effects of drugs on β-cells. We will in this paper describe the pursuit of novel β-cell neo-epitope biomarkers that could improve the clinical diagnosis and management of diabetes.

### Overview of T2DM

T2DM is a major cause of morbidity and mortality in the industrialized world, and cardiovascular complications affect as many as 50% of all T2DM patients [18]. The global prevalence of T2DM is rapidly increasing, and the number of people diagnosed with the disease worldwide has more than doubled over the past three decades, primarily due to a dramatic increase in number of obese people, as obesity is a major risk factor for development of T2DM [19]. Previously T2DM was considered a disease of the elderly, however, with increasing numbers of obese individuals of all ages, including children and adolescents, T2DM is now observed at all ages [19]. It is estimated that the prevalence of both type I and type II diabetes was 285 million in 2010, and this number is expected to increase to around 440 million by 2030, which represents 7.7% of the total adult population of the world aged 20–79 years [19]. Of the total population of diagnosed diabetics, at least 90% suffer from T2DM [19]. It is furthermore estimated that as many as 40-50% of people affected by T2DM or the stages preceding overt T2DM are undiagnosed [20].

### β-cell physiology and T2DM pathology

The human pancreas acts as both an exocrine and endocrine gland to aid the digestive system. The exocrine secretion of pancreatic juice into the duodenum aid the digestion of consumed nutrients. The pancreas also consists of highly vascularized and innervated endocrine mini-organs, called the islets of Langerhans, which make up 1-2% of the pancreatic mass, and which secrete several important hormones into the circulation. Each pancreatic islet includes at least five types of hormone-secreting cells (α-cells; β-cells; δ-cells; PP cells and ε-cells), of which the β-cell and α-cell secretes insulin and glucagon, respectively, and these pancreatic hormones are the dominant hormonal regulators of glucose metabolism. For a thorough review of pancreatic endocrine function we refer to the following reviews [21,22].

The β-cells constitute about 70-80% of pancreatic islet cells [23] and upon β-cell exposure to various stimuli including: [glucose, amino acids, free fatty acids, gastrointestinal hormones and neural stimuli], stored pro-insulin in granules is rapidly cleaved into insulin and C-peptide, which are released into the circulation. Amylin, which is also stored in the insulin storage granules, are co-secreted together with insulin, and accumulation of this protein in and around β-cells in states of insulin hypersecretion, may contribute to islet pathology as describe in the following section. A primary stimulus for insulin secretion is elevation of blood glucose above the fasting level, which is normally between 4.5-5.5mM. Insulin is an essential anabolic hormone that promotes sequestration of carbohydrate, fat and protein in storage depots throughout the body by exerting powerful actions principally on skeletal muscle, liver and adipose tissue. Insulin inadequacy, due to insufficient insulin production, as occur in T1DM, is characterized by lifelong dependency of exogenous insulin administration [24], as absence of insulin is correlated with impeded human survival.

**Figure 1:**
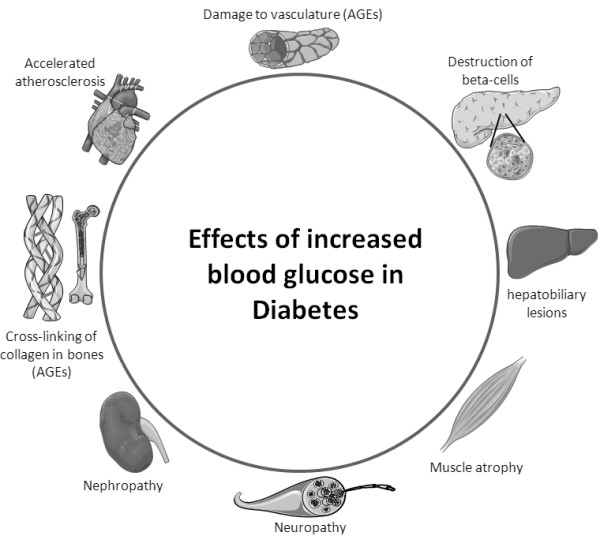
**Complications in T2DM related to increased blood glucose.** Elevated blood glucose can lead to the illustrated pathologies: destruction of β-cells, hepatobiliary lesions, muscle atrophy, neuropathy, nephropathy, formation of peripheral advanced glycation end-products (AGEs), accelerated atherosclerosis and damaged vasculature.

T2DM is a complex metabolic disease, characterized by elevated plasma glucose levels, loss of β-cell function and insulin resistance leading to disruption of the carbohydrate/lipid metabolism [25]. Decreased β-cell function and decreased insulin sensitivity in muscle, liver, pancreas and adipose tissue, result in elevated basal plasma glucose concentrations and/or impaired plasma glucose clearance after meal consumption [26]. The elevated plasma glucose is the cause of most clinical symptoms associated with T2DM such as compromised micro-vasculature, β-cell destruction, liver lesions, muscle atrophy, neuropathy, nephropathy, cross-linking of proteins by advanced glycation end-products (AGEs) causing fragile bones, and the most severe complication – accelerated artherosclerosis with increased risk of heart failure and stroke [26-28] (illustrated in Figure 1).

Before the onset of actual T2DM, patients can be diagnosed with impaired glucose tolerance or prediabetes, which are two pathological terms with great diagnostic overlap [19,29]. The diagnosis of T2DM has been recommended by both World Health Organization (WHO) and American Diabetes Association (ADA) to be established if HbA1c concentrations in blood are 6.5% (48mmol/mol) or higher [19]. For an introduction to HbA1c we recommend a review by Krishnamurti *et al.*[30].

**Figure 2:**
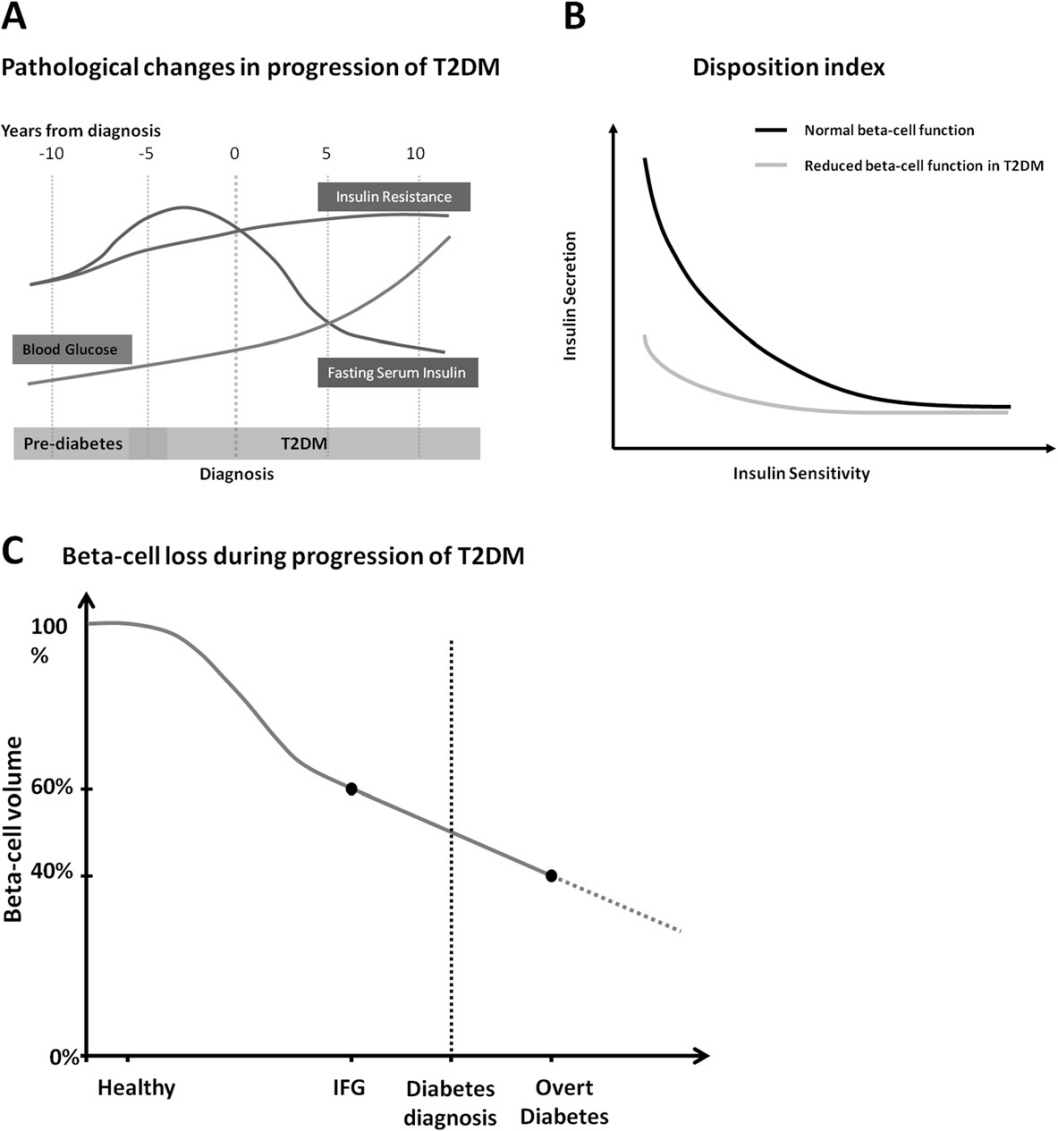
**Characteristics of T2DM development and progression. A**) Overview of changes in blood glucose, fasting serum insulin and insulin resistance during the initiation and progression of T2DM. Modified from [31]. **B**) Disposition index indicating the relationship between insulin sensitivity and insulin secretion in β-cells of normal individuals and T2DM patients. **C**) Β-cell loss during disease progression in T2DM. The graph is based on findings by Butler *et al.* and Holman *et al.*[6,34]. (IFG = Impaired fasting glucose).

An illustration of development of blood glucose, fasting serum insulin and insulin resistance during initiation and progression of T2DM is depicted in Figure 2A. Blood glucose gradually increases during initiation and progression of the disease, whereas insulin resistance primarily is increased before the diagnosis of T2DM [31]. Fasting serum insulin is actually increased in a period leading up to the diagnosis of T2DM, after which it will decline due to β-cell dysfunction and decreased β-cell mass [31]. It should be noted, however, that high fasting insulin levels (hyperinsulinemia) often occur at the same time as reduced insulin secretion upon glucose stimulation, due to β-cell dysfunction [32]. Furthermore, these two phenomena - elevated fasting insulin and reduced acute insulin secretion - are independent predictors of progression from normal to impaired glucose tolerance [32], which is a known risk factor for development of T2DM. The hyperinsulinemic state indicates that the β-cells are in constant overdrive, which leads to exhaustion of the β-cells, according to the overworked β-cell hypothesis [32,33]. It has been suggested that relieving the β-cells in the pre-diabetes state by inhibition of insulin secretion, would be a sensible way to protect the β-cells and thus lower the risk of development of diabetes [32].

In healthy individuals, insulin sensitivity and β-cell secretion of insulin are inversely and proportionally correlated. The product of these two parameters is constant and referred to as the disposition index [35]. For individuals developing T2DM this correlation is gradually lost, as the β-cells cannot meet the demand for excess insulin when insulin sensitivity is decreasing [4], as illustrated in Figure 2B. The relative contributions, to the progression of T2DM, of impaired insulin sensitivity and decreased β-cell function is controversial [1,4]. However, it appears that impaired β-cell function is a stronger predictor of development of T2DM than insulin resistance [1,4]. Being able to measure β-cell loss would be useful to assess the prognosis of T2DM in individual patients.

Treatments of T2DM should optimally reverse one or more of the pathological changes involved in T2DM, such as reducing the surplus blood glucose; normalizing islet function; improving fat tissue functionality and distribution; weight loss; and enhancing energy expenditure. Currently available treatments for T2DM include metformin, sulphonylureas, meglitinides, glitazones, glucagon-like peptide-1(GLP-1) analogues, dipeptidyl peptidase-4 (DPP-4) inhibitors, and insulin supplementation. While these in most cases are efficient in reducing fasting blood glucose and HbA1c levels, they are limited by a range of factors, such as loss of efficacy over time, intolerance, the need for injection, lack of effects on β-cells, and side effects including weight gain, hypoglycemia, fluid retention, heart failure, bone loss and others [19,36]. Thus, there is a continued search and need for novel treatments of T2DM. With novel tools to directly monitor β-cell function and life-span, new compounds which protect β-cell populations could be identified.

### The vulnerable β-cell

In several studies it has been shown that development of T2DM and progression is associated with loss of function of β-cells and a loss of β-cell mass [4,6,7,37]. Obese subjects with impaired fasting glucose (IFG), who are at increased risk of developing T2DM, display a 40% reduction in β-cell volume compared with non-diabetic obese controls [6]. Furthermore, it has been established that islet function is about 50% of normal at the time of T2DM diagnosis [34], and a reduction of 60% in β-cell volume has been established at necropsy of obese patients suffering from overt T2DM [4,6]. In addition, manifestation of pancreatic diabetes, in patients with underlying pancreatic disorders, appears when β-cell area has declined with approximately 65% [7]. The decline in β-cell mass was found to relate to increased β-cell apoptosis, whereas islet formation and β-cell replication remains normal in T2DM patients [4,6]. Figure 2C shows the decline in β-cell volume as reported in the literature. It should be mentioned, however, that a separate study identified a reduction in β-cell volume of only 24% in subjects with 1–5 years of overt diabetes [37]. Regardless of the precise reduction in β-cell volume and mass, several lines of evidence suggest that β-cell mass is progressively lost during initiation and progression of T2DM [4,6,7,37], and the development of T2DM is likely to be a combination of β-cell loss and β-cell functional deficiency [4,19,37].

**Figure 3:**
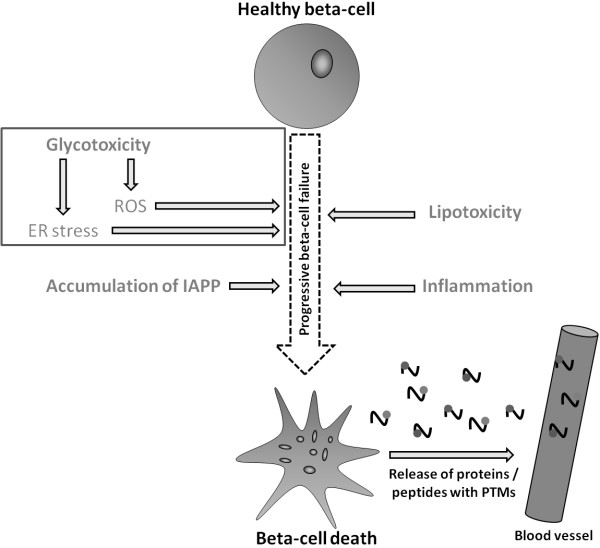
**Pathological processes leading to β-cell failure and death.** Several pathological processes can contribute to β-cell failure and β-cell death. These includes: Glucotoxicity, lipotoxicity, accumulation of islet amyloid polypeptide and inflammation. When β-cells become dysfunctional or undergo death, some β-cell proteins will undergo modifications by post translational modifications, and be released to circulation. (ER = endoplasmatic reticulum, IAPP = islet amyloid polypeptide, ROS = reactive oxygen species, PTMs = post translational modifications).

Several pathological processes lead to β-cell dysfunction and death, as illustrated in Figure 3. These include glucotoxicity, lipotoxicity, inflammation and amyloid deposition, which will be described briefly below. For a more elaborate review of these processes, we refer to Wajchenberg [4]. When β-cells undergo death or become dysfunctional, an array of proteins is expected to be modified by post translational modifications (PTMs) and released to circulation, as shown in Figure 3. Identifying such disease specific PTMs in circulation, could provide direct evidence of β-cell loss.

#### Glucotoxicity

Glucotoxicity is caused by prolonged exposure to supraphysiological concentrations of glucose, resulting in β-cell damage that may become irreversible [38,39]. Even transient exposure to supraphysiological glucose concentrations, as occurs in the pre-diabetes stage, might also have detrimental effects on β-cells [4,38], and could partially explain the observed decrease in β-cell mass before onset of T2DM.

Β-cells are by nature extremely sensitive to changes in glucose concentrations, and for this reason, prolonged exposure to high glucose concentrations is a major stress factor for these cells specifically. A high glucose concentration is a signal for high insulin production, which over time will impose a significant load on the endoplasmic reticulum (ER), eventually leading to ER stress (Figure 3), which has been shown to induce pro-apoptotic signals [4,40]. In addition to ER stress, high levels of glucose are known to induce oxidative stress by generation of reactive oxygen species (ROS) [38,39], as illustrated in Figure 3. ROS are highly damaging to β-cells, leading to β-cell dysfunction and even cell death [38,39]. Islet cells are in particular very vulnerable to ROS, as only low levels of antioxidant enzymes are expressed in the pancreatic islets [4,38,39].

#### Lipotoxicity

Experiments have shown that exposure of β-cells to high concentrations of free fatty acids (FFA) is not toxic to the cells when administered in combination with physiological glucose levels. However, high concentrations of FFA have been observed to have toxic effects on β-cells when glucose levels are elevated, indicating that lipotoxicity only occurs during hyperglycemia [4,39,41] (Figure 3).

In addition, lipoproteins have been shown to affect β-cells. Very low-density and low-density lipoproteins have been demonstrated to exert pro-apoptotic effects on β-cells, whereas high-density lipoproteins have been shown to protect against pro-apoptotic effects [42]. These findings demonstrate that the lipoprotein profile can contribute to disease pathogenesis and progression of β-cell failure [4,42].

#### Inflammation

Increased inflammatory mediators are common in T2DM (Figure 3). The inflammatory mediators are known to affect blood vessels and insulin-sensitive tissues, but evidence also suggests that inflammatory cytokines affect the β-cells directly by impairing their secretory function or inducing apoptosis [4]. An example is leptin, a pro-inflammatory cytokine produced by adipocytes, which has been shown to induce apoptosis in human β-cells after chronic exposure [4].

Β-cell apoptosis itself, induced by some of the several processes described here, is also indicated as a process that can provoke an auto-immune response, directed against the β-cells, as seen in “classical” T1DM [4].

#### Amyloid depositions

Depositions of amylin or islet amyloid polypeptide (IAPP) have been reported in up to 90% of T2DM patients, and it has for long been debated whether these depositions contribute to deterioration of β-cell function and ultimately β-cell death [4] (Figure 3). Depositions of IAPP around the β-cells occur as a consequence of uncorrected protein misfolding, caused by ER stress within the β-cells [43]. Soluble IAPP oligomers have been shown to exert toxic effects on β-cells and induce apoptosis, whereas stabilized IAPP depositions appear inert and non-toxic to the cells [6,44-47].

In states of insulin hyper-secretion, taking place prior to T2DM development (Figure 2A), high levels of amylin will also be secreted from the β-cells, which can contribute to amyloid deposition and thereby contribute to islet pathology in T2DM [6,43-47].

In summary, β-cells are vulnerable cells. Several pathological processes, as described above, progressively lead to decreased β-cell function, which manifests as decreased insulin synthesis and secretion, and can lead to β-cell failure and ultimately cell death [38,39], as illustrated in Figure 3. As β-cell dysfunction and death are some of the early initiating events, which over time can progress into development of T2DM, it would be valuable, with a simple and convenient test, to continuously assess β-cell functionality and β-cell death to better identify persons at risk of developing T2DM [2]. It should be possible to identify specific biomarkers which reflect the described changes inflicted on the β-cells, as β-cell failure and β-cell death will leave serological traits (Figure 3), which are currently unidentified.

### BIPED classification and the critical path initiative

The application of serological biomarkers in the clinic requires a high level of clinical validation and quantification. The BIPED (**B**urden of disease, **I**nvestigative, **P**rognostic, **E**fficacy of intervention and **D**iagnostic) classification of biomarkers was originally defined by the Osteoarthritis Biomarkers Network, a National Institutes of Health (NIH)-funded multidisciplinary group, but has since been adapted for other diseases, such as liver fibrosis [48,49]. The Burden of disease markers assesses the severity or extent of disease, typically at a single point in time, among individuals with a given disease (e.g. diabetes). The Investigative marker lacks sufficient information to allow for its inclusion in one of the other biomarker categories. The investigative category includes markers for which a relationship to various normal and abnormal biological processes has been identified, however has not yet been confirmed in human subjects. The key feature of a Prognostic marker is the ability to predict the future onset of disease among individuals without established disease or the progression of disease among those with the disease. Another application of prognostic markers is for prediction of response to treatment. An Efficacy-of-intervention biomarker provides information about the efficacy of treatment among persons with the disease. Diagnostic markers are defined by the ability to classify individuals as either having or not having a disease [48].

Another aspect of biomarker development is the awareness of their application. The US Food and Drug Administration (FDA) critical path initiative was launched in 2004 [50,51], with an overall objective of raising awareness about the cost of drug development and the relatively small number of drugs reaching FDA approval. Secondly, the critical path focused on the need for developing biomarkers, which could substantially shorten the time before important decisions were needed during drug development, such as ascertaining whether a drug’s effects would likely translate from animal to man, a rapid go-no-go decision following a phase I clinical study, narrowing of the dose-range from a phase II study, or predicting the right cohort before entering a phase III trial [16,51].

### Current markers in T2DM

As discussed earlier in this review, various tests are already used to diagnose and characterize T2DM. As defined in 2001 by the ‘Biomarkers Definitions Working Group’ [52] most of these tools can be characterized as biomarkers. Some have been in use for a long time, whereas others are recent discoveries and others are still subject to investigation. Current markers are described below, and summarized in Table 1, with indication of BIPED categories. The currently utilized biomarkers fall into the following BIPED categories: Burden of disease (B), Efficacy of intervention (E) and Diagnostic (D).

**Table 1:** Biomarkers used in relation to T2DM

**Biomarker**	**Target**	**Change in T2DM**	**Advantages**	**Disadvantages**	Examples of BIPED* classification
**HbA1C**	Blood glucose	Elevated	Easy and fast to measure.		**B, D**:
			No restrictions prior to measurement.		Used as the Gold standard for diagnosis and monitoring of T2DM [29]
					**E: HbA1C↓**
					Sulphonylureas+ Rosaglitazone [53]
					Prioglitazone [54]
					Balaglitazone and Pioglitazone [55]
					Liraglutide and Sitagliptin [56]
					DDP-IV inhibitor LC 15–0444 [57]
**Fasting plasma glucose (FPG)**	Blood glucose	Elevated	Easy and fast to measure.	Require patients to be fasting prior to sampling	**B, D**:
					Used in the diagnosis and monitoring of T2DM [29]
					**E: FPG ↓**
					Sulphonylureas+ Rosaglitazone [53]
					Prioglitazone [54]
					Balaglitazone and Pioglitazone [55]
					Liraglutide and Sitagliptin [56]
					DDP-IV inhibitor LC 15–0444 [57]
**Oral glucose tolerance test (OGTT) or Post-prandial glucose**	Blood glucose clearance	Glucose clearance: Impaired	OGTT: Accurate assessment of functional glucose clearance by liver or peripheral tissues	Two hour test.	**B, D**:
					Used in the diagnosis and monitoring of T2DM [29]
		Post prandial glucose: Elevated	Post-prandial glucose: A less time-consuming method to assess glucose clearance than OGTT	Time consuming test for the patient.	**E: Improved OGTT**
					Prioglitazone [54]
					**E: Post prandial glucose ↓**
					Balaglitazone and Pioglitazone [55]
**Pro-insulin**	Β-cell stress/dysfunction	Elevated	Only current marker to assess β-cell dysfunction	Usually combined with additional tests: Fasting insulin, C-peptide	**E: pro-insulin ↓**
**Split pro-insulin**			Proinsulin not directly influenced by therapeutic injections of insulin		Exenatide [58]
**Pro-insulin/Insulin ratio**					**E: Split pro-insulin ↓**
					Prioglitazone [54]
					**E: Split pro-insulin →**
					Gliclazide [54]
					**E: pro-insulin/insulin ratio ↓**
					Liraglutide and Sitagliptin [56]
					Exenatide [58]
**Fasting Insulin**	Β-cell functionality	Elevated in early stages of disease development.	Short half life of insulin	Fasting insulin levels changes with the stages of pathogenesis of T2DM	**E: Fasting Insulin ↑**
		Decreased in late stages of T2DM		Injections with insulin is used as treatment in T2DM	Gliclazide [54]
					Chlorpropamide [59]
					Glibenclamide [59]
					Insulin [59]
					**E: Fasting Insulin →**
					Exenatide [58]
					Liraglutide and Sitagliptin [56]
					**E: Fasting Insulin ↓**
					Sulphonylureas+ Rosaglitazone [53]
					Prioglitazone [54]
					Metformin [59]
**C-peptide**	Total insulin secretion	Elevated in early stages of disease development.	Half life: C-peptide		**E: C-peptide ↓**
		Decreased in late stages of T2DM	> Insulin. Improved assessment of total insulin secretion		Sulphonylureas+Rosaglitazone [53]
			C-peptide not directly influenced by therapeutic injections of insulin		Prioglitazone [54]
					DDP-IV inhibitor LC 15–0444 [57]
					**E: C-peptide ↑**
					Liraglutide and Sitagliptin [56]
					Gliclazide [54]

*BIPED (**B**urden of disease, **I**nvestigative, **P**rognostic, **E**fficacy of intervention, **D**iagnostic).

#### Glucose related biomarkers – FPG, OGTT and HbA1c

For decades, the gold standards for diagnosing diabetes were based on blood glucose levels, either FPG or the two-hour value in a standard OGTT [60]. Patients with a FPG ≥ 7.0 mmol/L or a 2h OGTT test value ≥ 11.1 mmol are considered diabetic. HbA1c is an alternative way of measuring the glucose level in circulation. HbA1c is a glycated version of hemoglobin located in the red blood cells. During the life-cycle of hemoglobin, which is approximately 3 months, a certain portion of the overall hemoglobin will be glycated, depending on the glucose level in blood. The HbA1c level is therefore proportional to average blood glucose concentration over the previous three months. In 2009, OGTT and FPG were replaced by HbA1c as the gold standard for disease diagnosis, with a T2DM diagnosis given at HbA1c concentrations of ≥ 6.5% [29] (equivalent to 48mmol/mol [61]). However, the ADA still recommends using a combination of HbA1c, FPG and OGTT for monitoring T2DM or T2DM risk assessment.

#### β-cell derived biomarkers – Pro-insulin, insulin and c-peptide

In healthy individuals, pro-insulin is initially stored in immature secretory vesicles in the β-cells, which bud off from the ER, and the protein is subsequently cleaved into insulin and c-peptide by endo-peptidases in the vesicle maturation process [62]. Pro-insulin secretion is low under normal conditions, but is found to be elevated in T2DM because it is not properly cleaved, either due to increased β-cell stress [63] or due to dysfunction in the secretory pathway of insulin [64]. The pro-insulin level is used as a marker of insulin resistance [65] and is often used in a pro-insulin/insulin ratio as an indicator of β-cell dysfunction [65,66].

Fasting insulin can be measured to assess basal β-cell productivity. In addition, insulin can be measured during OGTT to assess insulin response to glucose load. C-peptide is co-secreted with insulin in equivalent molar concentrations, but has a significantly longer half-life in the body than insulin; 20–30 min versus 3–5 min for insulin [67]. Due to a longer half-life combined with low hepatic retention, c-peptide is considered a more accurate measurement of endogenous insulin secretion than insulin itself [68].

Pro-insulin and c-peptide concentrations as well as pro-insulin/insulin ratios are currently being utilized in different Homeostasis Model Assessment (HOMA) models to estimate β-cell function/dysfunction (HOMA-B) and insulin sensitivity (HOMA-IS) [69]. Intact pro-insulin and pro-insulin/(insulin/c-peptide) ratio assessments are arguably the best current marker to assess β-cell stress/dysfunction and have previously been correlated insulin resistance and the progression of T2DM [70,71] and have also been demonstrated to have some predictive power to assess T2DM converters in the IRAS study [72,73]. However, its use to assess β-cell destruction/loss is only indirect and only useful in late stage T2DM as the β-cells by then have lost most of the redundancy found in the insulin secretory system. Novel biomarkers are needed that can be directly linked to β-cell loss before the secretory redundancy is lost and a biomarker that can directly determine whether the current level of β-cell stress/dysfunction causes the β-cell population to decline or not.

#### Additional T2DM related biomarkers

One approach to establishing prognostic biomarkers for T2DM is by combining of a broad spectrum of different known markers to form a prognostic algorithm for T2DM. This approach has been tested in several studies [74-76]. In addition, Wang *et al.* found that a panel of five amino acids have prognostic value in T2DM based on investigation of metabolite profiles in individuals who developed T2DM [77]. Adipokines are also an interesting class of molecules with potential to become biomarkers due to the intricate relationship between obesity and T2DM. Two recently discovered adipokines, chemerin and omentin-1, have been shown to be elevated or lowered in T2DM patients respectively [78], making these two adipokines potential new T2DM biomarkers. However, none of the biomarkers mentioned here have been fully validated and none is currently used for the general assessment of T2DM. These biomarkers can therefore at best only be characterized as Investigative (I) under the BIPED classification.

Despite the existing biomarkers, there is still a general lack of validated Prognostic (P) biomarkers for T2DM. Biomarkers reflecting β-cell loss could become valuable prognostic markers. In addition, such markers could also prove to be efficient markers for assessment of Efficacy of intervention (E), where the desired drug mode-of-action (MOA) is on the β-cells.

### Prior success with disease-specific post translational modifications

Neo-epitopes are post-translational modifications (PTMs) of proteins formed by processes such as protease cleavage, citrullination, nitrosylation, glycosylation, isomerisation and cross-linking [17]. Neo-epitopes are unique parts of a molecule that can be selected as a biochemical marker. Each protein modification results from a specific local physiological or pathological process [17]. Identifying neo-epitopes which are related to specific diseases can be visualized as finding specific protein fingerprints which relate to specific pathological changes.

The most commonly used neo-epitope biomarker in the field of diabetes is HbA1c, and as described in this paper, it is useful for monitoring response to treatment, as well as supporting the diagnosis of diabetes. However, changes in HbA1c levels occur slowly, and the magnitude of the changes is fairly small.

Another neo-epitope biomarker which has been used extensively is the bone resorption marker β-CTX-I. The use of this marker in the bone field has illustrated many of the benefits and a few of the challenges of this class of biomarkers [11].

**Figure 4:**
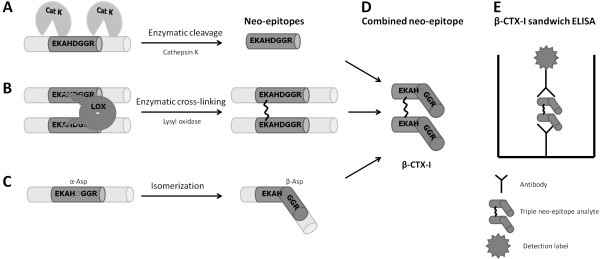
**Formation and measurement of β-CTX-I neo-epitopes. A**) Enzymatic cleavage of type I collagen by cathepsin K (Cat K) generates a cleavage specific noe-epitope. **B**) Enzymatic cross-linking of two type I collagen proteins with lysyl oxidase (LOX) generates a cross-linkede neo-epitope. **C**) Isomerization of aspatic acid (Asp) from α to β conformation generates an isomerized neo-epitope. **D**) Combined triple neo-epitope of β-CTX-I containing specific cleaveage, cross-linking and isomerized conformation. **E**) Illustration of sandwich ELISA for measurement of β-CTX-I.

In bone, the extracellular matrix (ECM) consists of 90% type I collagen, and this matrix is degraded by the bone-resorbing osteoclast [79-81]. The osteoclasts degrade type I collagen using the cysteine proteinase cathepsin K, and this has been shown to lead to the generation of the CTX-I fragment (^1207^EKAHDGGR^1214^) [79,82-84], as illustrated in Figure 4A. The CTX-I fragment hence contains a cathepsin K cleavage site as its primary neo-epitope (Figure 4A and D). However, in addition, it is a dipeptide linked together via a lysine crosslink adding another neo-epitope to the fragment [17,79], as seen in Figure 4B and D. Finally, it contains a DG amino acid sequence, and, with time, this site undergoes isomerisation with a conformational change of aspartic acid from α conformation to β conformation [17,79], as illustrated in Figure 4C. The β-CTX-I system measures the isomerized, hence aged form, whereas α-CTX-I measures the un-isomerized isoform. The isomerisation site thereby adds a third neo-epitope to this specific CTX fragment [17,79] (Figure 4C and D). Antibodies recognizing this specific fragment have been raised, and a sandwich ELISA measuring β-CTX-I specifically was developed [83-85], as illustrated in Figure 4E.

Studies of β-CTX-I have shown that it is elevated in post-menopausal women, and that is has predictive value for osteoporotic fractures. In addition it has been shown to respond to various anti-resorptive treatments, and to have predictive value for treatment efficacy on bone mineral density (BMD) [11]. Hence the β-CTX-I biomarker fits into all the BIPED categories [16]. This unique triple neo-epitope biomarker is used in both in vitro, preclinical and clinical studies within the bone field, as well as in studies of the bone safety of drugs related to other diseases for example in T2DM trials using thiazolidinediones (TZDs) [86].

### β-cell specific neo-epitopes as novel T2DM biomarkers

Currently the only direct way to assess β-cell mass and β-cell death in humans is taking biopsies of the pancreas [6,37], which is invasive and inconvenient to the patient. The indirect measures of β-cell function, levels of c-peptide and pro-insulin, are helpful but they cannot describe a decline in β-cell mass taking place in T2DM patients, whether before disease diagnosis or during disease progression. Therefore it is desirable to develop serological biomarkers reflecting directly the loss of β-cells.

**Figure 5:**
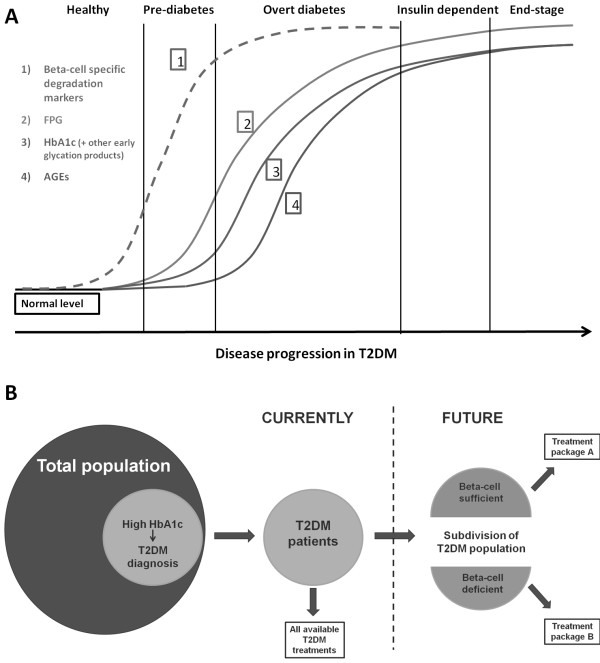
**Suggested biomarker progression during disease development and future segregation of T2DM patients. A**) Proposed biomarker development during T2DM initiation and progression. 1) Β-cell specific degradation markers, 2) Fasting plasma glucose (FPG), 3) HbA1c and other early glycation products, 4) Advanced glycation end-products (AGEs). **B**) Diagnosis of T2DM is based on an elevated concentration of HbA1c. Diagnosed T2DM patients can currently be offered all types of T2DM treatments. Future segregation of T2DM patients based on β-cell mass, could direct sub-groups of patients to more specific types of treatments.

It is suspected that the initiation of β-cell apoptosis, inflammation and islet remodeling, which are the processes responsible for decline in β-cell mass, precede the loss of function in glycemic control associated with T2DM [2]. The suggested relationship between β-cell specific degradation markers and the currently established biomarkers like FPG, HbA1c and AGEs, is illustrated in Figure 5A. During apoptosis and β-cell loss different proteases are involved in processing of the cells and components of the cells. With respect to proteases involved in processing of β-cell related proteins, previous studies have implicated matrix metalloproteinase 9 (MMP-9) and MMP-12, cathepsin B, and caspase 3 as being of high interest [9,14,87-90]. Rationales for their possible involvement in generation of β-cell biomarkers are given in Table 2. However, in relation to the proteases it is of importance that neither of these are specific for β-cells, and have been shown to be involved many different biological functions, unrelated to diabetes, and hence cannot on a individual level serve as biomarkers of β-cell loss. A description of these different processes is beyond the scope of this paper, and hence these will not be further discussed here. Hence, what raises the interest around these proteases is their known presence and activity in β-cells [9,14,87-90].

**Table 2:** Proteins and proteases of interest in development of β-cell specific biomarkers

***Target protein or Enzyme***		***Rationale***
**PROTEINS OF INTEREST**		
**Secretory Proteins**	**Insulin**	· Insulin is highly specific to the β-cells and is produced in high amounts.
		· Insulin degradation is a regulated process important for controlling insulin action by removing and inactivating the hormone.
		· Abnormalities in degradation of insulin are present in various pathological conditions including T2DM, and may be associated with development of clinical symptoms [91].
	**Amylin (IAPP)**	· Misfolding and deposit of IAPP is a major pathologic trait in a majority of T2DM patients [4].
		· IAPP oligomers have been demonstrated to be toxic to β-cells by inducing apoptosis [6,43-47].
		· Depositions of IAPP become a pathological extracellular matrix surrounding the β-cells, and degradation of this matrix could potentially serve as marker of developing T2DM.
**Β-cell Trans-membrane Proteins**	**Neuroligin-2, Neurexin 1α**	· Β-cell exocytic machinery is very similar to that of neuronal synapses, and for this reason the β-cells and neurons have some common traits [92].
		· It has been established that β-cells express specific proteins which are also found in the central nervous system (CNS), such as neuroligin-2 and neurexin-1 α [92].
		· As these proteins are rather specific to β-cells and neurons within the CNS, they might be suitable biomarker candidates for evaluation of β-cell degradation.
	**GLP-1 receptor, GIP receptor**	· The two incretin receptors GLP-1 receptor (GLP-1R) and GIP receptor (GIPR) are known to be expressed in pancreatic β-cells, but not exclusively by this cell type.
		· Activation of both GLP-1R and GIPR is known to stimulate insulin synthesis and insulin release [93,94], and both receptors have therefore been suggested as potential targets for the treatment of diabetes.
		· GLP-1R and GIPR have been demonstrated to form heterodimers, which could be of importance for fine-tuning incretin response [95].
		· Hyperglycemia has been found to lower the expression of both GLP-1R and GIPR, contributing to the diminished incretin action in hyperglycaemic states and diabetes [96,97].
	**GLUT1, GLUT2**	· Glucose transporters, GLUT1 and GLUT2, are important for the functionality of β-cells.
		· GLUT1 and GLUT2 are expressed in several tissues. However, neo-epitopes, which are specific to the pathological events involved with loss of β-cells, could be potential β-cell markers.
**T1DM Autoimmune Targets**	**GAD 65, IA-2, ZnT8**	· GAD65, IA-2 and ZnT8 are all established autoantigens in T1DM [98-100].
		· Autoantibodies directed against these autoantigens have also been identified in some T2DM patients [101].
		· It has been found that GAD65 is released during β-cell injury, and circulating GAD65 would therefore be a suitable marker for β-cell ill-health [2,102].
		· It has been established that measurements of GAD65 are able to detect β-cell death at a time point preceding the onset of hyperglycemia [2,102].
**PROTEASES OF INTEREST**		
**Caspase 3**		· Caspase 3 is a key enzyme in the enzymatic cascade initiating cell apoptosis.
		· Several pathological processes lead to β-cell apoptosis [4], rendering caspase 3 an interesting effector protease.
**MMP-12**		· MMP-12 is expressed primarily by macrophages and monocytes.
		· Β-cell loss can occur as consequence of local inflammation, and therefore, MMP-12 could be a protease of interest.
**MMP-9**		· MMP-9 is expressed primarily by macrophages and T-cells
		· Β-cell loss can occur as consequence of local inflammation, and therefore, MMP-9 could be a protease of interest.
**Cathepsin B**		· Cathepsin B is known to be present in pancreatic juice.
		· Cathepsin B has been speculated to be involved in the pathology of pancreatitis [103,104], and it could be hypothesized that similar mechanisms might, to some extent, be involved in development of T2DM.

It is very likely that one or more β-cell specific proteins are cleaved by proteases in pathological processes during disease initiation and progression when β-cells are lost, and therefore the combination of protease and protein could result in the generation of a β-cell specific, or at least selective, biomarker. These cleavages generate neo-epitopes of proteins some of which are released into circulation.

Neo-epitopes of β-cell proteins could be of potential interest for evaluation of β-cell health and death. Some of the potential protein candidates include: Insulin, IAPP (amylin), neurexin-1 α, neuroligin-2, gastric inhibitory polypeptide (GIP) receptor, glucagon-like peptide-1 (GLP-1) receptor, glucose transporter 1 and 2 (GLUT1, GLUT2), glutamic acid decarboxylase enzyme (GAD65), islet cell antigen 512 (IA-2) and zinc transporter 8 (ZnT8). In Table 2, this partial list of protein candidates is given, with rationales for the suitability of the chosen proteins as β-cell biomarkers.

To obtain an early diagnosis of diabetes we propose that β-cell loss can be detected by proteins derived directly from β-cells, preferably extracellular. A general issue for most of the suggested novel biomarkers is the somewhat lack of β-cell specificity, with insulin and amylin as exceptions. All of the mentioned proteins are highly expressed in β-cells, but also in other, often larger tissues. This could potentially mask any β-cell specific signal in the background noise coming from competing tissues. However the specificity could be obtained by the combination of high levels of expression in the pancreas together with a specific pattern of cleavage only observed from the pancreas. Proteins highly expressed in β-cells are likely to be cleaved and excreted into the bloodstream and the tissue- and disease specific protein fingerprinting approach described in the previous section could be applied, but it remains to be elucidated.

Amylin is co-secreted with insulin and is another hallmark secretory peptide hormone specific to pancreatic β-cells [105]. The emergence of IAPP plaques and the association with β-cell apoptosis [6,44] and β-cell specificity makes amylin a prime candidate for protein finger printing. However, amylin is readily degraded by insulin degrading enzyme (IDE) found highly expressed in kidney and muscle [106], which could make protease generated amylin fragments somewhat short lived.

Incretin hormones like GLP-1 and GIP have the ability to enhance insulin secretion and thereby improving clearance rate of glucose from the blood [93,94]. The direct effect is mediated by receptors expressed highly by the pancreatic β-cells. The receptors are also found in other tissues indicating an indirect effect e.g. from the nervous tissue [94]. Furthermore GLP-1 has shown anti-apoptotic effects and induction of neo-genesis in β-cells and insulin biosynthesis [94]. As the incretin receptors are expressed in β-cells these too are likely to be degraded upon β-cell disruption.

A similar fate could be attributed to other trans-membrane proteins such as GLUT1 and GLUT2, which are highly expressed in human and rodent pancreatic β-cells, respectively [107].

Neurons and β-cells have many similarities especially in the exocytotic machinery which in neurons is responsible for release of neurotransmitters and in β-cells mediate the release of insulin granules [108]. Proteins responsible for docking of vesicles and scaffolding proteins responsible for keeping the synapse tight is found both in neurons and β-cells [92]. Neurexin-1α and neuroligin-2 was identified in a systematic study of human tissue mRNAs designed to identify highly expressed, membrane-associated, human islet-specific proteins [109], and both proteins are involved in secretion of both neurotransmitters and insulin [110,111]. Neuroligin-2 and neurexin-1α are involved in vesicular docking and organizing the vesicles in the pre-synaptic area [110,111].

Another class of proteins of interest in relation to novel biomarkers are the targets of type I diabetes autoimmune antibodies and suggested potential biomarkers include glutamic acid decarboxylase enzyme (GAD65), islet cell antigen 512 (IA-2) and zinc transporter 8 (ZnT8) [2,98-100,102]. The specific destruction of pancreatic β-cells suggests high cell specificity even if the target proteins are expressed in other tissues as well. The proteins are ready available to the immune system and the surrounding tissues and could be potential targets for proteases released during ECM remodeling/destruction or by infiltrating macrophages. The autoimmune targets are either specific for β-cells or off-targets are otherwise protected (e.g. blood brain barrier).

To summarize, detection of any β-cell generated neo-epitopes in serum or plasma could prove valuable as biomarkers reflecting β-cell health and death aid our understanding of β-cell pathology in the initiation and progression of T2DM.

## Conclusions

It is not fully understood how β-cells, which play a critical role in both T1DM and T2DM, change during disease initiation and progression, and during drug interventions. This is primarily due to shortcomings in evaluation techniques.

We here suggest developing novel neo-epitope biomarkers, measurable in serum or plasma that will reflect β-loss and be used alongside existing markers for diabetes evaluation. Such novel biomarkers may segregate T2DM patients into appropriate treatment groups, based on the β-cell status of the individual patient, as illustrated in Figure 5B. Drug development in T2DM would also benefit from β-cell specific serological markers, as evaluation of β-cell health is suspected to become an important parameter, in future clinical trials.

## Abbreviations

ADA: American Diabetes Association; AGEs: Advanced glycation end-products; BIPED: Burden of disease, Investigative, Prognostic, Efficacy of intervention, Diagnostic; BMD: Bone mineral density; CNS: Central nervous system; DPP-4: Dipeptidyl peptidase-4; ECM: Extracellular matrix; ER: Endoplasmic reticulum; FDA: Food and Drug Administration; FFA: Free fatty acids; FPG: Fasting plasma glucose; GAD65: Glutamic acid decarboxylase enzyme; GIP: Gastric inhibitory polypeptide; GIPR: Gastric inhibitory polypeptide receptor; GLP-1: Glucagon-like peptide-1; GLP-1R: Glucagon-like peptide-1 receptor; GLUT1: Glucose transporter 1; GLUT2: Glucose transporter 2; GSH: Glutathione; HbA1c: Glycated haemoglobin A1c; HOMA: Homeostasis model assessment; IA-2: Islet cell antigen 512; IAPP: Islet amyloid polypeptide; IFG: Impaired fasting glucose; MMP: Matrix metalloproteinase; MOA: Mode-of-action; NIH: National Institutes of Health; OGTT: Oral glucose tolerance test; PTM: Post-translational modification; ROS: Reactive oxygen species; T1DM: Type I Diabetes Mellitus; T2DM: Type II Diabetes Mellitus; TZD: Thiazolidinedione; WHO: Wold Health Organization; ZnT8: Zinc transporter 8.

## Competing interests

Morten A. Karsdal owns stock options in Nordic Bioscience A/S. Anita Vibsig Neutzsky-Wulff, Kim Vietz Andreassen, Sara Toftegaard Hjuler, Michael J Feigh, Anne-Christine Bay-Jensen, Kim Henriksen and Qinlong Zheng are employed by Nordic Bioscience A/S, but own no stocks.

## Authors’ contributions

AVN and KVA have drafted the manuscript. STH, MF, AB, KH, QZ and MAK have all contributed substantially to the manuscript. All authors have read and approved the final manuscript.

## References

[B1] Gerich JE The genetic basis of type 2 diabetes mellitus: impaired insulin secretion versus impaired insulin sensitivity Endocr Rev 1998 19 491 503 10.1210/er.19.4.491 9715377

[B2] Hinke SA Finding GAD: early detection of beta-cell injury Endocrinology 2007 148 4568 4571 10.1210/en.2007-0861 17876036

[B3] Daneman D Type 1 diabetes Lancet 2006 367 847 858 10.1016/S0140-6736(06)68341-4 16530579

[B4] Wajchenberg BL beta-cell failure in diabetes and preservation by clinical treatment Endocr Rev 2007 28 187 218 17353295 10.1210/10.1210/er.2006-0038

[B5] Cnop M Welsh N Jonas JC Jorns A Lenzen S Eizirik DL Mechanisms of pancreatic beta-cell death in type 1 and type 2 diabetes: many differences, few similarities Diabetes 2005 54 Suppl 2 S97 S107 16306347 10.2337/diabetes.54.suppl_2.s97

[B6] Butler AE Janson J Bonner-Weir S Ritzel R Rizza RA Butler PC Beta-cell deficit and increased beta-cell apoptosis in humans with type 2 diabetes Diabetes 2003 52 102 110 10.2337/diabetes.52.1.102 12502499

[B7] Meier JJ Breuer TG Bonadonna RC Tannapfel A Uhl W Schmidt WE Schrader H Menge BA Pancreatic diabetes manifests when beta cell area declines by approximately 65% in humans Diabetologia 2012 55 1346 1354 10.1007/s00125-012-2466-8 22286529

[B8] Karsdal MA Delvin E Christiansen C Protein fingerprints - Relying on and understanding the information of serological protein measurements Clin Biochem 2011 44 1278 1279 10.1016/j.clinbiochem.2011.08.1135 21907191

[B9] Barascuk N Veidal SS Larsen L Larsen DV Larsen MR Wang J Zheng Q Xing R Cao Y Rasmussen LM A novel assay for extracellular matrix remodeling associated with liver fibrosis: An enzyme-linked immunosorbent assay (ELISA) for a MMP-9 proteolytically revealed neo-epitope of type III collagen Clin Biochem 2010 43 899 904 10.1016/j.clinbiochem.2010.03.012 20380828

[B10] Barascuk N Vassiliadis E Larsen L Wang J Zheng Q Xing R Cao Y Crespo C Lapret I Sabatini M Development and validation of an enzyme-linked immunosorbent assay for the quantification of a specific MMP-9 mediated degradation fragment of type III collagen–A novel biomarker of atherosclerotic plaque remodeling Clin Biochem 2011 44 900 906 10.1016/j.clinbiochem.2011.04.004 21549691

[B11] Henriksen K Leeming DJ Christiansen C Karsdal MA Use of bone turnover markers in clinical osteoporosis assessment in women: current issues and future options Womens Health (Lond Engl) 2011 7 689 698 10.2217/whe.11.74 22040210

[B12] Leeming DJ Bay-Jensen AC Vassiliadis E Larsen MR Henriksen K Karsdal MA Post-translational modifications of the extracellular matrix are key events in cancer progression: opportunities for biochemical marker development Biomarkers 2011 16 193 205 10.3109/1354750X.2011.557440 21506694

[B13] Veidal SS Vassiliadis E Bay-Jensen AC Tougas G Vainer B Karsdal MA Procollagen type I N-terminal propeptide (PINP) is a marker for fibrogenesis in bile duct ligation-induced fibrosis in rats Fibrogenesis Tissue Repair 2010 3 5 10.1186/1755-1536-3-5 20359335 PMC2860343

[B14] Veidal SS Karsdal MA Vassiliadis E Nawrocki A Larsen MR Nguyen QH Hagglund P Luo Y Zheng Q Vainer B MMP mediated degradation of type VI collagen is highly associated with liver fibrosis–identification and validation of a novel biochemical marker assay PLoS One 2011 6 e24753 10.1371/journal.pone.0024753 21935455 PMC3173456

[B15] Vassiliadis E Veidal SS Simonsen H Larsen DV Vainer B Chen X Zheng Q Karsdal MA Leeming DJ Immunological detection of the type V collagen propeptide fragment, PVCP-1230, in connective tissue remodeling associated with liver fibrosis Biomarkers 2011 16 426 433 10.3109/1354750X.2011.584131 21612338

[B16] Karsdal MA Henriksen K Leeming DJ Mitchell P Duffin K Barascuk N Klickstein L Aggarwal P Nemirovskiy O Byrjalsen I Biochemical markers and the FDA Critical Path: how biomarkers may contribute to the understanding of pathophysiology and provide unique and necessary tools for drug development Biomarkers 2009 14 181 202 10.1080/13547500902777608 19399662

[B17] Karsdal MA Henriksen K Leeming DJ Woodworth T Vassiliadis E Bay-Jensen AC Novel combinations of Post-Translational Modification (PTM) neo-epitopes provide tissue-specific biochemical markers–are they the cause or the consequence of the disease? Clin Biochem 2010 43 793 804 10.1016/j.clinbiochem.2010.03.015 20381482

[B18] World Health Organization Diabetes Fact sheet no 312 2008 [ http://www.who.int/mediacentre/factsheets/fs312/en/]

[B19] Nolan CJ Damm P Prentki M Type 2 diabetes across generations: from pathophysiology to prevention and management Lancet 2011 378 169 181 10.1016/S0140-6736(11)60614-4 21705072

[B20] Gakidou E Mallinger L Bbott-Klafter J Guerrero R Villalpando S Ridaura RL Aekplakorn W Naghavi M Lim S Lozano R Management of diabetes and associated cardiovascular risk factors in seven countries: a comparison of data from national health examination surveys Bull World Health Organ 2011 89 172 183 10.2471/BLT.10.080820 21379413 PMC3044248

[B21] Gromada J Franklin I Wollheim CB Alpha-cells of the endocrine pancreas: 35 years of research but the enigma remains Endocr Rev 2007 28 84 116 17261637 10.1210/er.2006-0007

[B22] Meglasson MD Matschinsky FM Pancreatic islet glucose metabolism and regulation of insulin secretion Diabetes Metab Rev 1986 2 163 214 10.1002/dmr.5610020301 2943567

[B23] In't VP Marichal M Microscopic anatomy of the human islet of Langerhans Adv Exp Med Biol 2010 654 1 19 10.1007/978-90-481-3271-3_1 20217491

[B24] Bluestone JA Herold K Eisenbarth G Genetics, pathogenesis and clinical interventions in type 1 diabetes Nature 2010 464 1293 1300 10.1038/nature08933 20432533 PMC4959889

[B25] Cho N Momose Y Peroxisome proliferator-activated receptor gamma agonists as insulin sensitizers: from the discovery to recent progress Curr Top Med Chem 2008 8 1483 1507 10.2174/156802608786413474 19075761

[B26] Kernan WN Inzucchi SE Viscoli CM Brass LM Bravata DM Horwitz RI Insulin resistance and risk for stroke Neurology 2002 59 809 815 10.1212/WNL.59.6.809 12349850

[B27] Day C Metabolic syndrome, or What you will: definitions and epidemiology Diab Vasc Dis Res 2007 4 32 38 10.3132/dvdr.2007.003 17469041

[B28] Wild S Byrne CD The role of treatment to increase HDL-cholesterol and decrease triglyceride concentrations in prevention of coronary heart disease in Type 2 diabetes Diabet Med 2004 21 Suppl 4 8 11 15315518 10.1111/j.1464-5491.2004.1424-5.x

[B29] American Diabetes A Standards of medical care in diabetes--2011 Diabetes Care 2011 34 Suppl 1 11 61 10.2337/dc11-0174 PMC3114493 21525493

[B30] Krishnamurti U Steffes MW Glycohemoglobin: a primary predictor of the development or reversal of complications of diabetes mellitus Clin Chem 2001 47 1157 1165 11427445

[B31] Leahy JJ The mechanisms of action for treatments of type 2 diabetes Diabetes Educ 2007 33 Suppl 5 101S 104S 17548896 10.1177/0145721707302S886

[B32] Hansen JB Arkhammar PO Bodvarsdottir TB Wahl P Inhibition of insulin secretion as a new drug target in the treatment of metabolic disorders Curr Med Chem 2004 11 1595 1615 10.2174/0929867043365026 15180566

[B33] Leahy JL Beta-Cell dysfunction with chronic hyperglycemia: the ''overworked beta-cell'' hypothesis Biabetes Revs 1996 4 298 319

[B34] Holman RR Assessing the potential for alpha-glucosidase inhibitors in prediabetic states Diabetes Res Clin Pract 1998 40 Suppl S21 S25 9740498 10.1016/s0168-8227(98)00038-2

[B35] Bergman RN Lilly lecture 1989. Toward physiological understanding of glucose tolerance. Minimal-model approach Diabetes 1989 38 1512 1527 10.2337/diabetes.38.12.1512 2684710

[B36] Krentz AJ Patel MB Bailey CJ New drugs for type 2 diabetes mellitus: what is their place in therapy? Drugs 2008 68 2131 2162 10.2165/00003495-200868150-00005 18840004

[B37] Rahier J Guiot Y Goebbels RM Sempoux C Henquin JC Pancreatic beta-cell mass in European subjects with type 2 diabetes Diabetes Obes Metab 2008 10 Suppl 4 32 42 18834431 10.1111/j.1463-1326.2008.00969.x

[B38] Robertson RP Harmon J Tran PO Tanaka Y Takahashi H Glucose toxicity in beta-cells: type 2 diabetes, good radicals gone bad, and the glutathione connection Diabetes 2003 52 581 587 10.2337/diabetes.52.3.581 12606496

[B39] Poitout V Robertson RP Glucolipotoxicity: fuel excess and beta-cell dysfunction Endocr Rev 2008 29 351 366 18048763 10.1210/er.2007-0023 PMC2528858

[B40] Song B Scheuner D Ron D Pennathur S Kaufman RJ Chop deletion reduces oxidative stress, improves beta cell function, and promotes cell survival in multiple mouse models of diabetes J Clin Invest 2008 118 3378 3389 10.1172/JCI34587 18776938 PMC2528909

[B41] Robertson RP Harmon J Tran PO Poitout V Beta-cell glucose toxicity, lipotoxicity, and chronic oxidative stress in type 2 diabetes Diabetes 2004 53 Suppl 1 S119 S124 14749276 10.2337/diabetes.53.2007.s119

[B42] Roehrich ME Mooser V Lenain V Herz J Nimpf J Azhar S Bideau M Capponi A Nicod P Haefliger JA Insulin-secreting beta-cell dysfunction induced by human lipoproteins J Biol Chem 2003 278 18368 18375 10.1074/jbc.M300102200 12594227

[B43] Hayden MR Tyagi SC Kerklo MM Nicolls MR Type 2 diabetes mellitus as a conformational disease JOP 2005 6 287 302 16006679

[B44] Butler AE Janson J Soeller WC Butler PC Increased beta-cell apoptosis prevents adaptive increase in beta-cell mass in mouse model of type 2 diabetes: evidence for role of islet amyloid formation rather than direct action of amyloid Diabetes 2003 52 2304 2314 10.2337/diabetes.52.9.2304 12941770

[B45] Janson J Ashley RH Harrison D McIntyre S Butler PC The mechanism of islet amyloid polypeptide toxicity is membrane disruption by intermediate-sized toxic amyloid particles Diabetes 1999 48 491 498 10.2337/diabetes.48.3.491 10078548

[B46] Westermark P Andersson A Westermark GT Islet amyloid polypeptide, islet amyloid, and diabetes mellitus Physiol Rev 2011 91 795 826 10.1152/physrev.00042.2009 21742788

[B47] Lorenzo A Razzaboni B Weir GC Yankner BA Pancreatic islet cell toxicity of amylin associated with type-2 diabetes mellitus Nature 1994 368 756 760 10.1038/368756a0 8152488

[B48] Bauer DC Hunter DJ Abramson SB Attur M Corr M Felson D Heinegard D Jordan JM Kepler TB Lane NE Classification of osteoarthritis biomarkers: a proposed approach Osteoarthr Cartil 2006 14 723 727 10.1016/j.joca.2006.04.001 16733093

[B49] Veidal SS Bay-Jensen AC Tougas G Karsdal MA Vainer B Serum markers of liver fibrosis: combining the BIPED classification and the neo-epitope approach in the development of new biomarkers Dis Markers 2010 28 15 28 20164543 10.3233/DMA-2010-0678 PMC3833336

[B50] Coons SJ The FDA's critical path initiative: a brief introduction Clin Ther 2009 31 2572 2573 10.1016/j.clinthera.2009.11.035 20110002

[B51] FDA rapport; Challenges and Opportunities Report - March 2004: Challenge and Opportunity on the Critical Path to New Medical Products http://www.fda.gov/downloads/ScienceResearch/SpecialTopics/CriticalPathInitiative/CriticalPathOpportunitiesReports/ucm113411.pdf]

[B52] Biomarkers Definitions Working Group Biomarkers and surrogate endpoints: preferred definitions and conceptual framework Clin Pharmacol Ther 2001 69 89 95 11240971 10.1067/mcp.2001.113989

[B53] Wolffenbuttel BH Gomis R Squatrito S Jones NP Patwardhan RN Addition of low-dose rosiglitazone to sulphonylurea therapy improves glycaemic control in Type 2 diabetic patients Diabet Med 2000 17 40 47 10.1046/j.1464-5491.2000.00224.x 10691158

[B54] Charbonnel BH Matthews DR Schernthaner G Hanefeld M Brunetti P A long-term comparison of pioglitazone and gliclazide in patients with Type 2 diabetes mellitus: a randomized, double-blind, parallel-group comparison trial Diabet Med 2005 22 399 405 15787663 10.1111/j.1464-5491.2004.01426.x

[B55] Henriksen K Byrjalsen I Qvist P Beck-Nielsen H Hansen G Riis BJ Perrild H Svendsen OL Gram J Karsdal MA Efficacy and safety of the PPARgamma partial agonist balaglitazone compared with pioglitazone and placebo: a phase III, randomized, parallel-group study in patients with type 2 diabetes on stable insulin therapy Diabetes Metab Res Rev 2011 27 392 401 10.1002/dmrr.1187 21328517

[B56] Pratley RE Nauck M Bailey T Montanya E Cuddihy R Filetti S Thomsen AB Sondergaard RE Davies M Liraglutide versus sitagliptin for patients with type 2 diabetes who did not have adequate glycaemic control with metformin: a 26-week, randomised, parallel-group, open-label trial Lancet 2010 375 1447 1456 10.1016/S0140-6736(10)60307-8 20417856

[B57] Rhee EJ Lee WY Yoon KH Yoo SJ Lee IK Baik SH Kim YK Lee MK Park KS Park JY A multicenter, randomized, placebo-controlled, double-blind phase II trial evaluating the optimal dose, efficacy and safety of LC 15–0444 in patients with type 2 diabetes Diabetes Obes Metab 2010 12 1113 1119 10.1111/j.1463-1326.2010.01303.x 20977584

[B58] Buse JB Henry RR Han J Kim DD Fineman MS Baron AD Effects of exenatide (exendin-4) on glycemic control over 30 weeks in sulfonylurea-treated patients with type 2 diabetes Diabetes Care 2004 27 2628 2635 10.2337/diacare.27.11.2628 15504997

[B59] United Kingdom Prospective Diabetes Study Group United Kingdom Prospective Diabetes Study 24: a 6-year, randomized, controlled trial comparing sulfonylurea, insulin, and metformin therapy in patients with newly diagnosed type 2 diabetes that could not be controlled with diet therapy Ann Intern Med 1998 128 165 175 9454524 10.7326/0003-4819-128-3-199802010-00001

[B60] American Diabetes A American Diabetes Association Standards of medical care in diabetes--2010 Diabetes Care 2010 33 Suppl 1 11 61

[B61] Jones GR Barker G Goodall I Schneider HG Shephard MD Twigg SM Change of HbA1c reporting to the new SI units Med J Aust 2011 195 45 46 21728944 10.5694/j.1326-5377.2011.tb03190.x

[B62] Steiner DF Park SY Stoy J Philipson LH Bell GI A brief perspective on insulin production Diabetes Obes Metab 2009 11 Suppl 4 189 196 19817801 10.1111/j.1463-1326.2009.01106.x

[B63] Leahy JL Halban PA Weir GC Relative hypersecretion of proinsulin in rat model of NIDDM Diabetes 1991 40 985 989 10.2337/diabetes.40.8.985 1860563

[B64] Porte D Jr Kahn SE Hyperproinsulinemia and amyloid in NIDDM. Clues to etiology of islet beta-cell dysfunction? Diabetes 1989 38 1333 1336 10.2337/diabetes.38.11.1333 2695369

[B65] Mykkanen L Zaccaro DJ Hales CN Festa A Haffner SM The relation of proinsulin and insulin to insulin sensitivity and acute insulin response in subjects with newly diagnosed type II diabetes: the Insulin Resistance Atherosclerosis Study Diabetologia 1999 42 1060 1066 10.1007/s001250051271 10447516

[B66] Haffner SM Mykkanen L Valdez RA Stern MP Holloway DL Monterrosa A Bowsher RR Disproportionately increased proinsulin levels are associated with the insulin resistance syndrome J Clin Endocrinol Metab 1994 79 1806 1810 10.1210/jc.79.6.1806 7989488

[B67] Marques RG Fontaine MJ Rogers J C-peptide: much more than a byproduct of insulin biosynthesis Pancreas 2004 29 231 238 10.1097/00006676-200410000-00009 15367890

[B68] Polonsky KS Rubenstein AH C-peptide as a measure of the secretion and hepatic extraction of insulin. Pitfalls and limitations Diabetes 1984 33 486 494 10.2337/diabetes.33.5.486 6373457

[B69] Wallace TM Levy JC Matthews DR Use and abuse of HOMA modeling Diabetes Care 2004 27 1487 1495 10.2337/diacare.27.6.1487 15161807

[B70] Pfutzner A Kann PH Pfutzner AH Kunt T Larbig M Weber MM Forst T Intact and total proinsulin: new aspects for diagnosis and treatment of type 2 diabetes mellitus and insulin resistance Clin Lab 2004 50 567 573 15481632

[B71] Pfutzner A Pfutzner AH Larbig M Forst T Role of intact proinsulin in diagnosis and treatment of type 2 diabetes mellitus Diabetes Technol Ther 2004 6 405 412 10.1089/152091504774198124 15198846

[B72] Loopstra-Masters RC Haffner SM Lorenzo C Wagenknecht LE Hanley AJ Proinsulin-to-C-peptide ratio versus proinsulin-to-insulin ratio in the prediction of incident diabetes: the Insulin Resistance Atherosclerosis Study (IRAS) Diabetologia 2011 54 3047 3054 10.1007/s00125-011-2322-2 21959959

[B73] Hanley AJ D'Agostino R Jr Wagenknecht LE Saad MF Savage PJ Bergman R Haffner SM Increased proinsulin levels and decreased acute insulin response independently predict the incidence of type 2 diabetes in the insulin resistance atherosclerosis study Diabetes 2002 51 1263 1270 10.2337/diabetes.51.4.1263 11916954

[B74] Goldfine AB Gerwien RW Kolberg JA O'Shea S Hamren S Hein GP Xu XM Patti ME Biomarkers in fasting serum to estimate glucose tolerance, insulin sensitivity, and insulin secretion Clin Chem 2011 57 326 337 10.1373/clinchem.2010.156133 21149503 PMC4274783

[B75] Kolberg JA Jorgensen T Gerwien RW Hamren S McKenna MP Moler E Rowe MW Urdea MS Xu XM Hansen T Development of a type 2 diabetes risk model from a panel of serum biomarkers from the Inter99 cohort Diabetes Care 2009 32 1207 1212 10.2337/dc08-1935 19564473 PMC2699726

[B76] Salomaa V Havulinna A Saarela O Zeller T Jousilahti P Jula A Muenzel T Aromaa A Evans A Kuulasmaa K Thirty-one novel biomarkers as predictors for clinically incident diabetes PLoS One 2010 5 e10100 10.1371/journal.pone.0010100 20396381 PMC2852424

[B77] Wang TJ Larson MG Vasan RS Cheng S Rhee EP McCabe E Lewis GD Fox CS Jacques PF Fernandez C Metabolite profiles and the risk of developing diabetes Nat Med 2011 17 448 453 10.1038/nm.2307 21423183 PMC3126616

[B78] El-Mesallamy HO El-Derany MO Hamdy NM Serum omentin-1 and chemerin levels are interrelated in patients with Type 2 diabetes mellitus with or without ischaemic heart disease Diabet Med 2011 28 1194 1200 10.1111/j.1464-5491.2011.03353.x 21668495

[B79] Leeming DJ Henriksen K Byrjalsen I Qvist P Madsen SH Garnero P Karsdal MA Is bone quality associated with collagen age? Osteoporos Int 2009 20 1461 1470 10.1007/s00198-009-0904-3 19330423

[B80] Neutzsky-Wulff AV Sorensen MG Kocijancic D Leeming DJ Dziegiel MH Karsdal MA Henriksen K Alterations in osteoclast function and phenotype induced by different inhibitors of bone resorption–implications for osteoclast quality BMC Musculoskelet Disord 2010 11 109 10.1186/1471-2474-11-109 20515459 PMC2891608

[B81] Teitelbaum SL Bone resorption by osteoclasts Science 2000 289 1504 1508 10.1126/science.289.5484.1504 10968780

[B82] Henriksen K Leeming DJ Byrjalsen I Nielsen RH Sorensen MG Dziegiel MH Martin TJ Christiansen C Qvist P Karsdal MA Osteoclasts prefer aged bone Osteoporos Int 2007 18 751 759 10.1007/s00198-006-0298-4 17216130

[B83] Bonde M Qvist P Fledelius C Riis BJ Christiansen C Immunoassay for quantifying type I collagen degradation products in urine evaluated Clin Chem 1994 40 2022 2025 7955372

[B84] Garnero P Ferreras M Karsdal MA Nicamhlaoibh R Risteli J Borel O Qvist P Delmas PD Foged NT Delaisse JM The type I collagen fragments ICTP and CTX reveal distinct enzymatic pathways of bone collagen degradation J Bone Miner Res 2003 18 859 867 10.1359/jbmr.2003.18.5.859 12733725

[B85] Bonde M Qvist P Fledelius C Riis BJ Christiansen C Applications of an enzyme immunoassay for a new marker of bone resorption (CrossLaps): follow-up on hormone replacement therapy and osteoporosis risk assessment J Clin Endocrinol Metab 1995 80 864 868 10.1210/jc.80.3.864 7883844

[B86] Zinman B Haffner SM Herman WH Holman RR Lachin JM Kravitz BG Paul G Jones NP Aftring RP Viberti G Effect of rosiglitazone, metformin, and glyburide on bone biomarkers in patients with type 2 diabetes J Clin Endocrinol Metab 2010 95 134 142 10.1210/jc.2009-0572 19875477

[B87] Newby AC Matrix metalloproteinase inhibition therapy for vascular diseases Vascul Pharmacol 2012 56 232 244 10.1016/j.vph.2012.01.007 22326338

[B88] Tomita T Immunocytochemical localisation of caspase-3 in pancreatic islets from type 2 diabetic subjects Pathology 2010 42 432 437 10.3109/00313025.2010.493863 20632819

[B89] Mueller-Steiner S Zhou Y Arai H Roberson ED Sun B Chen J Wang X Yu G Esposito L Mucke L Antiamyloidogenic and neuroprotective functions of cathepsin B: implications for Alzheimer's disease Neuron 2006 51 703 714 10.1016/j.neuron.2006.07.027 16982417

[B90] Stutzer I Esterhazy D Stoffel M The pancreatic beta cell surface proteome Diabetologia 2012 55 1877 1889 10.1007/s00125-012-2531-3 22460761 PMC3369137

[B91] Duckworth WC Bennett RG Hamel FG Insulin degradation: progress and potential Endocr Rev 1998 19 608 624 10.1210/er.19.5.608 9793760

[B92] Suckow AT Comoletti D Waldrop MA Mosedale M Egodage S Taylor P Chessler SD Expression of neurexin, neuroligin, and their cytoplasmic binding partners in the pancreatic beta-cells and the involvement of neuroligin in insulin secretion Endocrinology 2008 149 6006 6017 10.1210/en.2008-0274 18755801 PMC2613060

[B93] Fujita Y Wideman RD Asadi A Yang GK Baker R Webber T Zhang T Wang R Ao Z Warnock GL Glucose-dependent insulinotropic polypeptide is expressed in pancreatic islet alpha-cells and promotes insulin secretion Gastroenterology 2010 138 1966 1975 10.1053/j.gastro.2010.01.049 20138041

[B94] Holst JJ Deacon CF Vilsboll T Krarup T Madsbad S Glucagon-like peptide-1, glucose homeostasis and diabetes Trends Mol Med 2008 14 161 168 10.1016/j.molmed.2008.01.003 18353723

[B95] Schelshorn D Joly F Mutel S Hampe C Breton B Mutel V Lutjens R Lateral allosterism in the glucagon receptor family: glucagon-like Peptide 1 induces g-protein-coupled receptor heteromer formation Mol Pharmacol 2012 81 309 318 10.1124/mol.111.074757 22108912

[B96] Yamada Y Seino Y Physiology of GIP–a lesson from GIP receptor knockout mice Horm Metab Res 2004 36 771 774 10.1055/s-2004-826162 15655707

[B97] Xu G Kaneto H Laybutt DR Duvivier-Kali VF Trivedi N Suzuma K King GL Weir GC Bonner-Weir S Downregulation of GLP-1 and GIP receptor expression by hyperglycemia: possible contribution to impaired incretin effects in diabetes Diabetes 2007 56 1551 1558 10.2337/db06-1033 17360984

[B98] Vaziri-Sani F Delli AJ Elding-Larsson H Lindblad B Carlsson A Forsander G Ivarsson SA Ludvigsson J Marcus C Lernmark A A novel triple mix radiobinding assay for the three ZnT8 (ZnT8-RWQ) autoantibody variants in children with newly diagnosed diabetes J Immunol Methods 2011 371 25 37 10.1016/j.jim.2011.06.011 21708156 PMC3170126

[B99] Reijonen H Daniels TL Lernmark A Nepom GT GAD65-specific autoantibodies enhance the presentation of an immunodominant T-cell epitope from GAD65 Diabetes 2000 49 1621 1626 10.2337/diabetes.49.10.1621 11016444

[B100] Hoppu S Harkonen T Ronkainen MS Simell S Hekkala A Toivonen A Ilonen J Simell O Knip M IA-2 antibody isotypes and epitope specificity during the prediabetic process in children with HLA-conferred susceptibility to type I diabetes Clin Exp Immunol 2006 144 59 66 10.1111/j.1365-2249.2006.03033.x 16542366 PMC1809627

[B101] Hawa MI Fava D Medici F Deng YJ Notkins AL De MG Leslie RD Antibodies to IA-2 and GAD65 in type 1 and type 2 diabetes: isotype restriction and polyclonality Diabetes Care 2000 23 228 233 10.2337/diacare.23.2.228 10868836

[B102] Waldrop MA Suckow AT Marcovina SM Chessler SD Release of glutamate decarboxylase-65 into the circulation by injured pancreatic islet beta-cells Endocrinology 2007 148 4572 4578 10.1210/en.2006-1367 17584960

[B103] Kukor Z Mayerle J Kruger B Toth M Steed PM Halangk W Lerch MM Sahin-Toth M Presence of cathepsin B in the human pancreatic secretory pathway and its role in trypsinogen activation during hereditary pancreatitis J Biol Chem 2002 277 21389 21396 10.1074/jbc.M200878200 11932257

[B104] Lerch MM Halangk W Human pancreatitis and the role of cathepsin B Gut 2006 55 1228 1230 10.1136/gut.2006.092114 16905693 PMC1860045

[B105] Moore CX Cooper GJ Co-secretion of amylin and insulin from cultured islet beta-cells: modulation by nutrient secretagogues, islet hormones and hypoglycemic agents Biochem Biophys Res Commun 1991 179 1 9 10.1016/0006-291X(91)91325-7 1679326

[B106] Bennett RG Duckworth WC Hamel FG Degradation of amylin by insulin-degrading enzyme J Biol Chem 2000 275 36621 36625 10.1074/jbc.M006170200 10973971

[B107] Yang H Wright JR Jr Human beta cells are exceedingly resistant to streptozotocin in vivo Endocrinology 2002 143 2491 2495 10.1210/en.143.7.2491 12072379

[B108] Burgoyne RD Morgan A Secretory granule exocytosis Physiol Rev 2003 83 581 632 12663867 10.1152/physrev.00031.2002

[B109] Maffei A Liu Z Witkowski P Moschella F Del PG Liu E Herold K Winchester RJ Hardy MA Harris PE Identification of tissue-restricted transcripts in human islets Endocrinology 2004 145 4513 4521 10.1210/en.2004-0691 15231694

[B110] Mosedale M Egodage S Calma RC Chi NW Chessler SD Neurexin-1alpha contributes to insulin-containing secretory granule docking J Biol Chem 2012 287 6350 6361 10.1074/jbc.M111.299081 22235116 PMC3307300

[B111] Suckow AT Zhang C Egodage S Comoletti D Taylor P Miller MT Sweet IR Chessler SD Transcellular neuroligin-2 interactions enhance insulin secretion and are integral to pancreatic beta cell function J Biol Chem 2012 287 19816 19826 10.1074/jbc.M111.280537 22528485 PMC3370167

